# Optimization of the Alkaline Pretreatment of Rice Straw for Enhanced Methane Yield

**DOI:** 10.1155/2013/968692

**Published:** 2012-12-24

**Authors:** Zilin Song, Gaihe Yang, Xinhui Han, Yongzhong Feng, Guangxin Ren

**Affiliations:** ^1^College of Forestry, Northwest A&F University, Yangling, Shaanxi Province 712100, China; ^2^Research Center for Recycling Agricultural Engineering Technology of Shaanxi Province, Yangling, Shaanxi Province 712100, China; ^3^College of Agronomy, Northwest A&F University, Yangling, Shaanxi Province 712100, China

## Abstract

The lime pretreatment process for rice straw was optimized to enhance the biodegradation performance and increase biogas yield. The optimization was implemented using response surface methodology (RSM) and Box-Behnken experimental design. The effects of biodegradation, as well as the interactive effects of Ca(OH)_2_ concentration, pretreatment time, and inoculum amount on biogas improvement, were investigated. Rice straw compounds, such as lignin, cellulose, and hemicellulose, were significantly degraded with increasing Ca(OH)_2_ concentration. The optimal conditions for the use of pretreated rice straw in anaerobic digestion were 9.81% Ca(OH)_2_ (w/w TS), 5.89 d treatment time, and 45.12% inoculum content, which resulted in a methane yield of 225.3 mL/g VS. A determination coefficient (*R* ^2^) of 96% was obtained, indicating that the model used to predict the anabolic digestion process shows a favorable fit with the experimental parameters.

## 1. Introduction

The anaerobic digestion (AD) of animal manure and agricultural byproducts has drawn increased attention because the aforementioned materials serve as valuable alternatives to fossil fuels as energy sources; AD can also destroy pathogenic organisms and reduce the problems associated with organic waste disposal [1]. A major source of biogas production is agricultural byproducts, such as manure and deep litter, harvest residues, and energy crops [2]. As the largest agricultural country in the world, China has abundant biomass resources, including crop straw, firewood, agricultural residues, and organic wastes [3]. According to the report of the Agriculture Ministry of China, about 710 million tons of crop straw is produced in China each year. Among them, lignocellulosic biomass, such as rice straw (RS), corn stalks, and wheat straw, account fors 79.5% of total production [4]. Processing these agricultural residues can generate 55.8 million m^3^ of biogas and resolve the energy shortage in rural areas [5]. The effective use of biomass also considerably alleviates environmental problems. Therefore, developing a cost-effective technology for biogas production from lignocellulosic biomass is important for rural China.

AD generally involves four steps: hydrolysis, acidogenesis, acetogenesis, and methanogenesis. Biological hydrolysis is identified as the rate-limiting step because the components of lignocellulosic biomass (e.g., lignin) that interlinks cellulose and hemicellulose layers are difficult to degrade [6]. Pretreating agricultural residues by mechanical size reduction, heat treatment, chemical treatment, and biological treatment is usually implemented during hydrolysis to improve digestibility [7]. Heat pretreatment increases biodegradability, but the thermal process consumes a more substantial amount of energy than that used up in chemical methods. In biological pretreatment, a high biodegradability rate is realized at exorbitant costs [8]. Thus, most researchers deem alkali pretreatment as the best method for enhancing the biodegradation of complex materials; it presents the most significant benefits [9–14]. The preferred chemical, Ca(OH)_2_, has been extensively used in improving biodegradability because of its low cost, high recoverability, safe handling, and minor environmental effects [15]. Chang et al. [16] reported that the optimal lime pretreatment conditions for the AD of poplar wood are 150°C, 6 h, and 0.1 g of Ca(OH)_2_/g of dry biomass. Rabelo et al. [17] revealed that the highest production of methane was observed in pretreatments with the highest solid concentration of lime; that is, 8%, a value that is 32% and 56% higher than that of 6% and 4% lime, respectively. Kaar and Holtzapple [10] showed that pretreatment with slake lime increased the enzymatic hydrolysis of corn stover to nine times higher than that generated by untreated corn stover. The recommended pretreatment conditions are lime loading at 0.075 g (per g of dry biomass), water loading of 5 g (g dry biomass), and heating for 4 h at 120°C. In their previous study on fallen leaves, Liew et al. [7] demonstrated that the greatest enhancement in methane yield was achieved at an S/I ratio (substrate/inoculum) of 6.2 with a NaOH loading of 3.5%, which is 24-fold higher than that of the control (without NaOH addition). These results suggest that pretreatment time, temperature, chemical concentration, and inoculums amount have more considerable effects on the digestibility of agricultural wastes.

Response surface methodology (RSM) is considered a useful method for analyzing several independent parameters on response to expected dependent variables because it integrates mathematical and statistical techniques [18]. RSM is more practical than theoretical models because it is grounded on experimental methods and ultimately expresses the overall effects of parameters on processes on the basis of the interactive effect of variables; this methodology therefore presents important applications in experiment design and process optimization [19]. In the last few years, RSM has been applied in optimizing and evaluating the interactive effects of independent factors of numerous chemical and biochemical processes involved in AD [20]. However, little information has been provided on using the RSM model to analyze the effect of hydrolysis pretreatment on biogas production with agricultural residues. 

To address this problem, this work aims to study the effect of three operating parameters—pretreatment time, concentration of pretreatment chemicals, and inoculum amount—on the AD of RS under batch conditions. A Box-Behnken experimental design (BBD) combined with RSM was applied to determine the effect of these operating parameters on methane yield.

## 2. Materials and Methods

Experiments were conducted in a self-designed, constant-temperature anaerobic fermentation equipment, without pH adjustment of pretreated RS. A Box-Behnken experimental matrix was designed to evaluate the effect and interactions of Ca(OH)_2_ concentration (LC), pretreatment time (PT), and inoculum amount (IA), as well as to determine the conditions that enable the derivation of the highest biogas production rates and self-stability at mesophilic temperature.

### 2.1. Material Collection

RS was collected from a rice field near Northwest A&F University (Yangling, Shaanxi, China). The RS was cut into 20–30 mm (length) pieces by using a grinder. The characteristics of RS are shown in Table 1. The inoculum was obtained from the 8 m^3^ hydraulic biogas digester in a model village powered by household biogas (Yangling, Shaanxi, China). The total solid (TS) content of the slurry was 5.1%.

### 2.2. Experimental Setup

The concentration of Ca(OH)_2_ used in this study was chosen on the basis of previous research [10, 21]. 4%, 8%, and 12% of Ca(OH)_2_ were added to beakers that contain 500 g RS in treated straw, and the ratio of solid to liquid was 1 : 3. Finally, all the prepared beakers were covered with plastic films secured with a plastic ring and then stored in a chamber at ambient temperature ((25 ± 2)°C) for 3, 7, and 11 days, respectively. At the end of the pretreatment, 50 g of each pretreated RC was sampled and dried after washing in an electronic oven (HengFeng SFG-02.600, Huangshi, China) at 80°C for 48 h. The samples were then kept in a 4°C refrigerator for composition determination. After pretreatment, the RS was directly used for AD without additional treatment.

**Figure 1:**
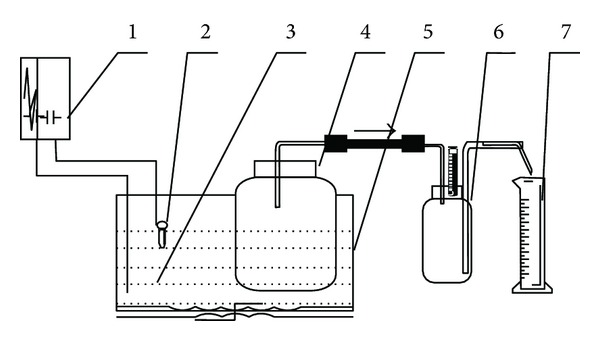
Controllable and constant-temperature anaerobic fermentation device. 1: Relay; 2: temperature controller; 3: heater; 4: anaerobic digester; 5: trough at constant temperature; 6: receiver; 7: measuring cylinder.

A laboratory-scale simulated experiment using a self-designed, constant-temperature anaerobic fermentation equipment was then conducted [22]. The equipment consists of an anaerobic digester (1 L Erlenmeyer flask), a biogas collector (1 L Erlenmeyer flask), and a 1 L measuring cylinder used to measure the water discharged from the collector (Figure 1). The digesters were placed in a constant-temperature water bath (water-heated, 1°C fluctuation) with a temperature controller that displays temperature. The biogas generated in the digester was transported into the headspace of a bottle by using a glass pipe. The water in the bottle was pressed out and injected into the receiver through another glass pipe. The volume of water discharged from the bottle represents the volume of biogas generated in the digester.

Each sample of pretreated RS was used as the digestion material, with the untreated RS as the control. Each treatment was performed in triplicate. The volume of each digester was 1 L, with a 0.75 L working volume. The fermentation lasted for 41 d. The digestion material and inocula were added to each digester (the total weight of digestion material was 700 g), and deionized water was added as necessary to obtain 8% TS content [23]. Moreover, the anaerobic fermentation was set at 37°C to eliminate the effect of the inocula, and 140, 245, and 350 g of the slurry were digested. 

### 2.3. Experiment Design

BBDs are a class of rotatable or nearly rotatable second-order designs based on three-level incomplete factorial designs. The number of experiments (*N*) required for the development of a BBD is defined as *N* = 2*k*(*k* − 1) + *C*_0_, (where *k* is the number of factors and *C*_0_ denotes the number of central points) [24]. To describe the interactive effects of LC, PT, and IA on the responses, methane production with RS was optimized using the BBD. Seventeen continuous-flow experiments were conducted as the LC, PT, and IA of RS were selected as independent variables at ranges of 4%–12% (w/w), 3–7 d, and 20%–50% (w/w), respectively. These ranges were selected on the basis of previous research [7, 10] and on the basis of the values used in our previous work. The experimental design was analyzed by RSM. The coded levels and corresponding real values of the operating variables considered in the experimental design are summarized in Table 2.

### 2.4. Analysis and Calculations

The daily biogas production of each anaerobic digester was recorded using the water displacement method. The corresponding cumulative biogas volume was also calculated. Methane content in the produced biogas was analyzed with a fast methane analyzer (Model DLGA-1000, Infrared Analyzer, Dafang, Beijing, China). The TS, volatile solids (VSs), pH, and alkalinity of the materials were determined using the standard water and wastewater examination methods [25]. Organic carbon content and total nitrogen were analyzed using the Walkley-Black [26] and Kjeldahl methods [27], respectively. Volatile fatty acid (VFA) was analyzed by the colorimetric method [28], and the results were indicated as acetic acid expressions. Cellulose, hemicellulose, and lignin contents were analyzed following the method of Lin et al. [8].

## 3. Results and Discussion

### 3.1. Effect of Pretreatments on the Main Compositions of Rice Straw

Table 3 shows the hemicelluloses, cellulose, and lignin contents in RS pretreated with Ca(OH)_2_. The cellulose and hemicellulose contents in ground RS remarkably decreased in all the chemical treatments compared with the control. These results are consistent with previous studies, in which 30% to 60% of cellulose and hemicellulose were decomposed in the anaerobic biogasification of alkaline-treated materials [11]. Some researchers showed that PT considerably affected the degradation of the main components of agricultural wastes and that long treatments increased the reduction in cellulose and hemicellulose contents under certain conditions. Kim and Holtzapple [29] reported that cellulose in corn stover exhibited an insignificant effect when degraded at a duration of 4 weeks at 35°C. After 8 weeks, however, 89.8% of the cellulose fragments were degraded and only 10.2% of the cellulose existed as intact glucooligomers. The current work showed similar results: under all LCs, the 11 d pretreatment exhibited higher reduction in cellulose and hemicelluloses than did the 3 and 7 d pretreatments. At the same PT, a high LC resulted in a high decrease in cellulose and hemicelluloses. These results indicate that a high pretreatment concentration more effectively breaks down the lignocellulose matrix and changes its chemical components. The high Ca(OH)_2_ considerably changed the microstructure of the cell wall and increased the accessibility of contents to anaerobic microorganisms, facilitating the use of soluble compounds with low molecular weights by microorganisms and increasing biodegradability. 

The solubility of hemicellulose during lime pretreatment is closely related to delignification [29]. Similar to a high concentration of cellulose and hemicellulose, the high LC and long pretreatment increased lignin degradation. This finding is inconsistent with that of Xu et al.'s study [12], in which increased calcium loading significantly decreased lignin reduction under different treatment conditions. This discrepancy can be attributed to different agronomic crops and growth conditions. In the current study, lime pretreatment led to limited lignin reduction that ranged from 4.4% to 24.3%, a value considerably lower than those generated by other alkaline chemicals, such as NaOH and NH_3_·H_2_O [13, 30]. Reduced lignin reduction may have resulted from the formation of a calcium-lignin complex. Calcium ions, which carry two positive charges, tend to crosslink negatively charged lignin molecules under alkaline conditions. This crosslinking is caused by the ionization of functional groups, such as carboxyl, methoxyl, and hydroxyl, through the formation of stoichiometric bonds, thereby weakening lignin solubility by Ca(OH)_2_ during pretreatment processes [31].

### 3.2. Effect of Pretreatments on Daily Methane Production

**Figure 2:**
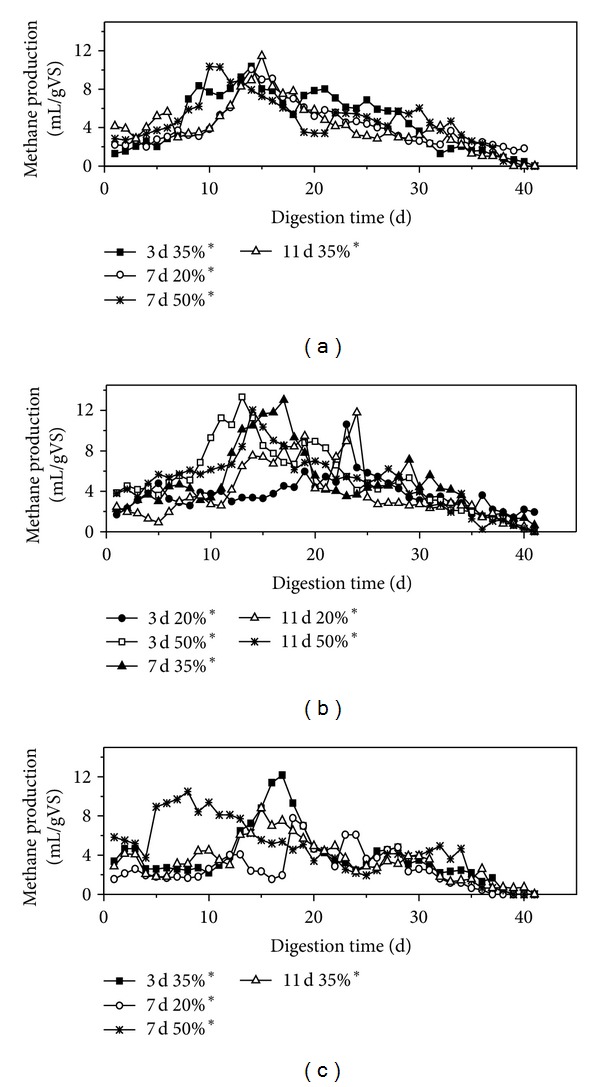
Daily  methane production for each pretreatment of rice straw. (a) 4% Ca(OH)_2_ pretreatment; (b) 8% Ca(OH)_2_ pretreatment; (c) 12% Ca(OH)_2_ pretreatment.

In the BBD, 17 runs of anaerobic fermentation were conducted with RS. The daily methane production is depicted in Figure 2, which shows only 13 treatments because 5 of 17 runs in the experimental design were central points (8% Ca(OH)_2_, 7 d PT, and 35% IA). Thus, the mean values of the runs were derived when the daily methane production was analyzed. The same method was used to analyze the VFA/ALK ratio and pH. Similar trends of daily methane production were found in all the treatments. Methane was generated after seeding, and it kept increasing as the peak value was reached. Methane production exhibited several small peaks before production finally ceased. However, the peak value of methane production and the time spent reaching the peak value differed for each treatment. On days 8–14, the IA of 50% pretreatment reached the methane peak value earlier than did the IAs of 20% and 35% pretreatments. The highest peak values of methane yield for the 4%, 8%, and 12% pretreatments (11.46, 13.33, 12.17 mL/g VS) were obtained at an 11 d treatment with IA 35%, a 3 d treatment with IA 50%, and a 3 d treatment with IA 35%, respectively. The results showed that a certain increase in IA improved the biodigestibility of RC, facilitating its use by anaerobic microorganisms, thereby requiring less time for digestion. Furthermore, the peak value of the pretreatments increased as LC increased but subsequently declined, indicating that a high LC can provide more organic matter to anaerobic microorganisms for biogas generation. Excessive Ca^2+^ may be toxic for anaerobic fermentation. The daily methane production also fluctuated considerably for the 8% and 12% treatments, as indicated by the appearance of several small peaks. This phenomenon may be related to the dynamic balance between the metabolism of acidogene and methanogen in AD [14]. The concentration of 8% and 12% Ca(OH)_2_ more effectively broke down the lignocellulose matrix and supplied sufficient organic matter for methanogen, thereby hastening the growth of methanogens and increasing methane yield. The shortage of organic acid as it gradually decreased in the substrate constrained methanogen activity, but stimulated acidogene, causing a drop in methane yield. The alternate activities of methanogen and acidogene resulted in the appearance of a peak in methane production. However, the peak value in methane production decreased because the substrate concentration was lower than the initial concentration.

### 3.3. Effect of Pretreatments on pH Values, Total Volatile Fatty Acids, and Alkalinity

**Figure 3:**
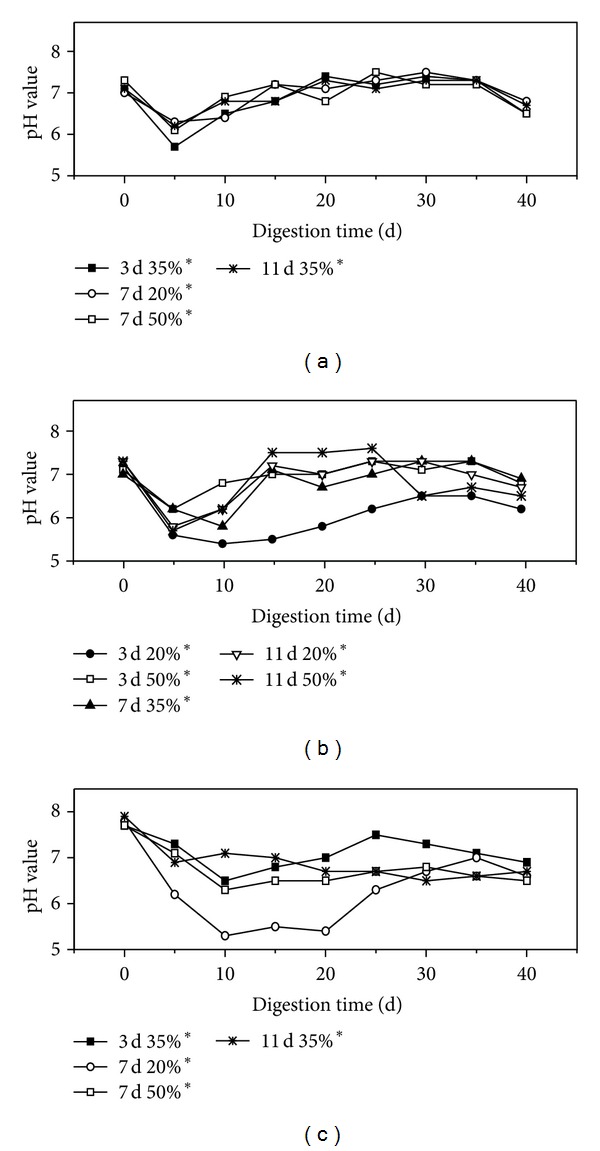
pH value for each pretreatment of rice straw. (a) 4% Ca(OH)_2_ pretreatment; (b) 8% Ca(OH)_2_ pretreatment; (c) 12% Ca(OH)_2_ pretreatment.

Imbalances in hydrolytic, fermentative, acetogenic, and methanogenic functions during AD can result in low methane yield and digestion failure [7]. The accumulation of VFA can significantly decrease pH value, subsequently restraining methanogen and disrupting the AD process. Thus, pH and total VFA are always considered key indicators of AD performance [32].  Figure 3 shows the changes in pH during the 41 d AD. The initial pH values of all the pretreatments, which ranged from 7.0 to 8.0, were above the operational pH of 7.0 recommended by Lahav and Morgan [32]. The pretreatments exhibited similar pH curves, showing an initial decreasing trend followed by an increase thereafter. This result can be explained by the interaction between acidogene and methanogen activities during AD [14]. In the initial AD stage, the sufficient substrate provided resources for acidogene. The availability of resources increased the growth of acidogenes and production of acid compounds, such as organic acids, H_2_S, H_2_, and NH_3_, thereby resulting in a rapid decline in pH value. As the AD continues, the decrease in substrate constrained acidogene activity; the accumulation of organic acids provided the resources for methanogen, thereby generating alkalines in the form of carbon dioxide, ammonia, and bicarbonate [33] and increasing the pH value. At 12% Ca(OH)_2_, pH value remained at a value lower than that observed in the other pretreatments during AD at an IA of 20% for 7 d at 4% and for 11 d at 8%; these findings are possibly related to the higher Ca(OH)_2_ concentration that result in a greater biodegradable acid soluble compounds with low molecular weights and making the pH value of 12% Ca(OH)_2_ lower.

**Figure 4:**
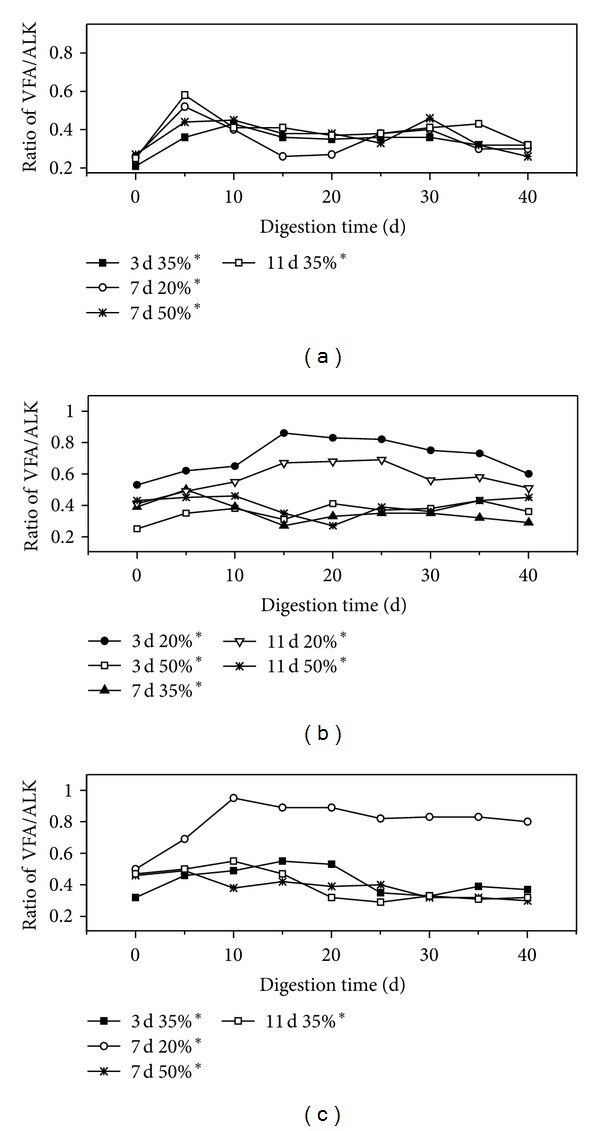
Ratio of volatile fatty acids to alkalinity for each pretreatment of rice straw. (a) 4% Ca(OH)_2_ pretreatment; (b) 8% Ca(OH)_2_ pretreatment; (c) 12% Ca(OH)_2_ pretreatment.

At a high buffering capacity, pH value is an ineffective measure of the stability of an anaerobic process. Small changes in pH occur under considerable changes in process performance [34]. The stability criterion for AD is always indicated by the ratio of total VFA to buffering capacity measured as alkalinity [35]. Although the optimal total VFA/alkalinity ratio of each AD reactor is unique, a ratio of 0.3–0.5 is generally regarded as optimal for liquid AD, and a ratio that exceeds 0.6 is regarded as indicative of overfeeding [7]. As fermentation proceeds, the VFA/alkalinity ratio of all the pretreatments initially increased and then stabilized (Figure 4). The VFA/alkalinity ratio of IA of 20% for 7 d at 12% Ca(OH)_2_, 20% IA for 3 d, and 11 d at 8% Ca(OH)_2_ was higher than 0.6 after 10–15 d; this was different from other pretreatments during AD ranging from approximately 0.3 to 0.5. This result is attributed to the lower activity of methanogenic bacteria in the 20% IA pretreatment, which reduced the buffering capacity in the fermentation environment. The VFA/alkalinity ratio in the initial stage of AD was generally high because acidogene produced considerably more VFA; as the AD progressed, the VFA/alkalinity ratio stabilized and then subsequently dropped because of the high alkalinity caused by methanogen.

### 3.4. Modeling and Optimization of Methane Production of Ca(OH)_2_-Pretreated Rice Straw

Box-Behnken analysis enabled the optimization of the effects of PT, LC, and IA, thereby achieving maximal methane production (Table 2). The results in Table 2 were used to run ANOVA and multiple regression analysis in the Design-Expert software. The polynomial model equation was also used. This approach enabled the prediction of the optimum degree of methane yield and its corresponding optimum variables.  Table 4 shows the ANOVA results for the fitness and degree of significance of the model and its variables. Regression analysis produced the following quadratic equation (1), which had a high regression coefficient (*R* ^2^ = 0.96):

(1) Y=−32.09511+21.63398×X1+14.62316×X2+5.71086×X3+0.11892×X1X2+0.26431×X1X3−0.074696×X2X3−2.22220×X12−1.04312×X22−0.078660×X32,

where *X*_1_, *X*_2_, and *X*_3_ represent the LC, PT, and IA, respectively, and *Y* denotes the methane yield. The significance of each variable is shown in Table 4; the *F*-value of the model was 18.86 with a very low probability (*P*) value of 0.0004. These imply a significant effect on the process of methane production. The significance of the linear, quadratic, and interactive terms of the model was verified in the same manner. The results revealed that in the AD of pretreated RC, IA (*X*_3_) is the most significant factor, followed by the LC (*X*_1_). The “lack of fit” with a *P* value of 0.9199 showed that it is nonsignificant, implying that the model exhibits fitness for prediction. 

The results of ridge analysis suggested that the optimal conditions were *X*_1_ = 9.81 (LC), *X*_2_ = 5.89 (PT), *X*_3_ = 45.12 (IA), which resulted in a predicted *Y* (methane yield) of 217.82 mL/g VS RS of the theoretical maximum in (1), thereby increasing methane yield by about 67.55%. 

To determine the validity of the predicted value, five experiments were performed at optimal conditions. For easy operation, the conditions were adjusted to 9.8% LC, 6 d PT, and 45% IA. The experiments yielded a methane production of 225.3 mL/g VS (compared with untreated RS).

### 3.5. Analysis of Response Surfaces

**Figure 5:**
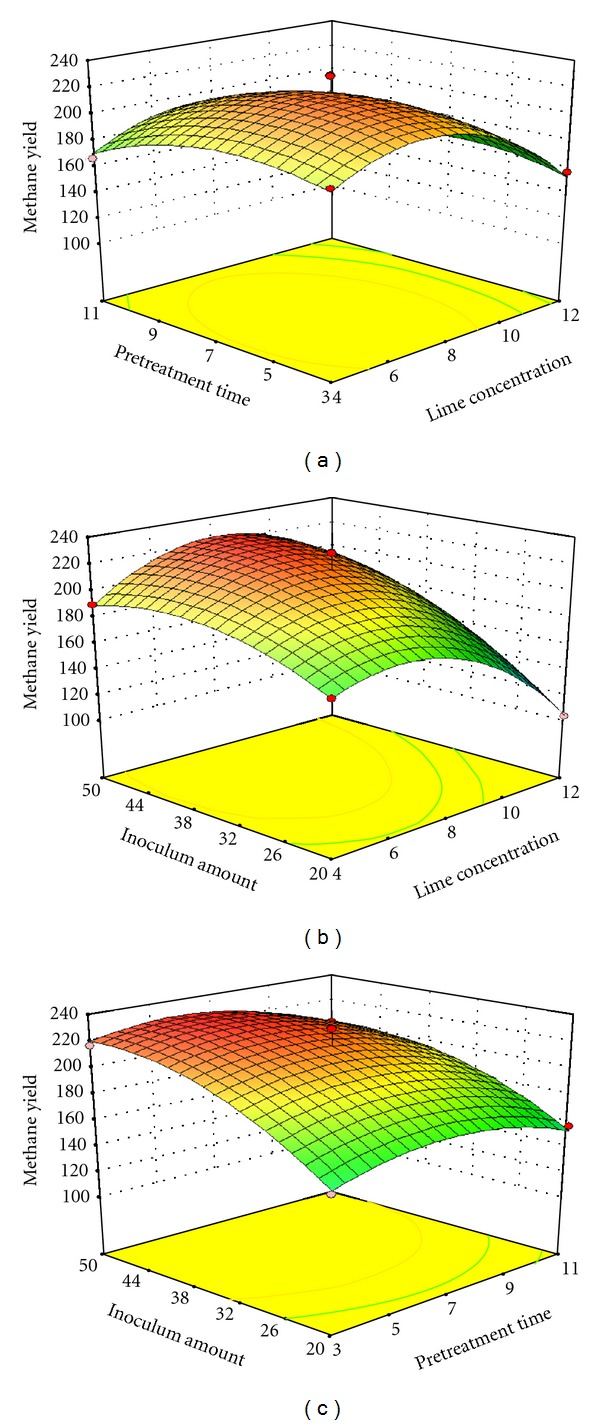
Effects of Ca(OH)_2_ concentration, treatment time, and inoculum amount on the AD of pretreated rice straw. (a) Interactive effect of Ca(OH)_2_ concentration and pretreatment time; (b) interactive effect of Ca(OH)_2_ concentration and inoculum amount; (c) interactive effect of pretreatment time and inoculum amount.

The experimental results were visualized in three-dimensional response surface plots that show the correlation between two parameters and methane yield.  Figure 5(a) shows the response surface plot of the LC and PT effects on the methane yield of pretreated RS at an IA of 35%. Increased LC and PT resulted in increased methane yield, but a further increase reduced methane production. The combined effect of LC and IA on the daily methane yield is depicted in Figure 5(b). IA and LC showed significant interaction. At a PT of 7 d, the highest methane yield was produced at a high IA and medium-level LC; a high LC may decrease methane yield with increasing IA. These findings agree with those of Liew et al. [7], who achieved a highest methane yield of 81.8 L/kg VS at 3.5% NaOH loading during a 30 d digestion; in their study, increasing the NaOH loading from 3.5% to 5.0% decreased methane yield.  Figure 5(c) illustrates the effects of PT and IA on the daily methane yield. The optimum methane yield is achieved at high IA and low pretreatment time, with the linear effect of IA being dominant. The comparison of the three plots shows that IA more significantly affected the methane production after pretreatment during the digestion process, as presented by a precipitous curve.

## 4. Conclusion 

Under Ca(OH)_2_ pretreatment, the improvement of methane production of rice straw highly depended on pretreatment variables such as LC, PT, and IA of AD. The modeling and optimization of the disintegration techniques employed in this research confirmed that the optimum degree of pretreated RS for anaerobic fermentation by alkaline disintegration was 225.3 mL/g VS at optimal variables of 9.81% Ca(OH)_2_ (w/w TS), 5.89 d PT, and 45.12% IA. A determination coefficient (*R* ^2^) of 96% was obtained, indicating that the model used to predict the anabolic digestion process exhibits a favorable fit with the experimental parameters. The analysis of response surfaces indicated that at the PT of 7 d, the highest methane yield was produced at a high IA and medium-level LC, and a high LC may decrease methane yield with increasing IA. For the three parameters, IA was more significant factor affecting the methane production than after pretreatment during the digestion process.

## Figures and Tables

**Table 1:** Characteristics of rice straw.

Parameters	Values (%)
Total solids (TSs)	95.8 ± 3.6
Volatile solid (VS)	84.0 ± 4.3 (of TS)
Total carbon (TC)	33.96 ± 1.87
Total nitrogen (TN)	0.49 ± 0.040
Carbon-to-nitrogen (C/N) ratio	69.31 ± 3.64

Result = mean ± standard deviation (SD).

**Table 2:** Box-Behnken design for the Ca(OH)_2_ pretreatment of rice straw.

Run	Coded			Uncoded			Response
	LC (%)	PT (d)	IA (%)	LC (%)	PT (d)	IA (%)	Methane yield (mL/g VS)
1	0	−1	−1	8	3	20	154.0
2	0	1	1	8	11	50	200.0
3	0	0	0	8	7	35	200.0
4	1	−1	0	12	3	35	156.5
5	0	0	0	8	7	35	215.1
6	1	0	1	12	7	50	190.0
7	0	1	−1	8	11	20	155.9
8	0	0	0	8	7	35	207.1
9	1	0	−1	12	7	20	103.6
10	0	0	0	8	7	35	228.2
11	0	0	0	8	7	35	229.0
12	−1	0	−1	4	7	20	167.0
13	−1	0	1	4	7	50	190.0
14	−1	1	0	4	11	35	167.0
15	1	1	0	12	11	35	141.1
16	−1	−1	0	4	3	35	190.0
17	0	−1	1	8	3	50	216.1

LC: Ca(OH)_2_ concentration; PT: pretreatment time; IA: inoculum amount.

**Table 3:** Changes in the main compositions of corn straw after chemical pretreatment (%).

	Control	4% Ca(OH)_2_			8% Ca(OH)_2_			12% Ca(OH)_2_		
		3 d	7 d	11 d	3 d	7 d	11 d	3 d	7 d	11 d
Cellulose	45.36 ± 0.55	43.72 ± 0.86	40.98 ± 0.65	39.82 ± 1.00	37.29 ± 1.04	31.07 ± 0.49	29.74 ± 0.79	31.61 ± 1.13	22.14 ± 0.86	20.97 ± 0.58
Hemicellulose	28.14 ± 0.43	26.16 ± 1.02	22.51 ± 0.88	21.1 ± 0.91	19.81 ± 0.57	13.99 ± 1.14	11.54 ± 0.37	13.91 ± 0.39	11.24 ± 0.88	9.98 ± 0.64
Lignin	6.88 ± 0.09	6.58 ± 0.06	6.45 ± 0.10	6.23 ± 0.07	6.36 ± 0.03	6.22 ± 0.09	6.17 ± 0.04	6.39 ± 0.08	5.36 ± 0.15	5.21 ± 0.08

Result = mean ± SD.

**Table 4:** ANOVA for the response surface quadratic model.

Source	Statistics				
	Sum of squares	df	Mean square	*F*-value	Prob > *F*
Model	17714.58	9	1968.29	18.86	0.0004
*X*_1_	1885.66	1	1885.66	18.07	0.0038
*X*_2_	345.74	1	345.74	3.31	0.1115
*X*_3_	5807.45	1	5807.45	55.66	0.0001
*X*_1_ *X*_2_	14.48	1	14.48	0.14	0.7205
*X*_1_ *X*_3_	1005.94	1	1005.94	9.64	0.0172
*X*_2_ *X*_3_	80.34	1	80.34	0.77	0.4093
*X*_1_^2^	5322.85	1	5322.85	51.01	0.0002
*X*_2_^2^	1172.85	1	1172.85	11.24	0.0122
*X*_3_^3^	1318.90	1	1318.90	12.64	0.0093
Residual	730.41	7	104.34	—	—
Lack of fit	76.94	3	25.65	0.16	0.9199
Pure error	653.47	4	163.37	—	—
Cor Total	18444.99	16	—	—	—

## Abbreviations

AD: Anaerobic digestion
BBD: Box-Behnken experimental design
LC: Ca(OH)_2_ concentration
IA: Inoculum amount
PT: Pretreatment time
RS: Rice straw
RSM: Response surface methodology
TS: Total solids
VS: Volatile solid
TC: Total carbon
TN: Total nitrogen
C/N: Carbon-to-nitrogen ratio.
